# ECPR In Penetrating Chest Trauma With Thoracic Aortic Injury: A Case Report

**DOI:** 10.1002/ccr3.72194

**Published:** 2026-04-03

**Authors:** Xiang Gao, Linzi Jin, Yan Li

**Affiliations:** ^1^ Department of Emergency Medicine, Shanghai Fourth People's Hospital, School of Medicine Tongji University Shanghai China

**Keywords:** cardiac arrest, case report, ECMO, ECPR, electrical impedance tomography, extracorporeal cardiopulmonary resuscitation, penetrating chest trauma, thoracic aortic injury, VA‐ECMO

## Abstract

Cardiac arrest due to severe chest trauma with thoracic aortic injury presents a significant challenge in emergency medicine. Extracorporeal cardiopulmonary resuscitation (ECPR) offers a potentially lifesaving intervention for such cases. We report a case of a 22‐year‐old male who sustained an open chest trauma with thoracic aortic injury from a stab wound, resulting in cardiac arrest. The patient was treated with ECPR combined with emergent surgical repair. Electrical impedance tomography (EIT) monitoring provided real‐time functional assessment of regional lung mechanics during the 8‐day ECMO support period. Serial chest radiographs documented improvement following bedside hematoma evacuation, with improvement in PaO_2_/FiO_2_ ratio from 118 to 442.5. The patient achieved successful ECMO weaning; however, due to prolonged cardiac arrest time exceeding 30 min, neurological recovery was not achieved (GCS remained 3 T), and the family withdrew care after 2 months. Despite the unfavorable neurological outcome, this case demonstrates the technical feasibility of ECPR in severe penetrating chest trauma and provides valuable experience for managing similar cases with potentially shorter arrest times.

## Key Clinical Message

In selected cases of penetrating chest trauma with cardiac arrest, extracorporeal cardiopulmonary resuscitation (ECPR) combined with resuscitative thoracotomy may provide a viable rescue option when hemorrhage is controlled but cardiac function does not recover. Despite poor neurological outcomes in prolonged arrests, early ECPR initiation offers the potential for hemodynamic stabilization and may improve survival in similar cases with shorter downtime.

## Graphical abstract

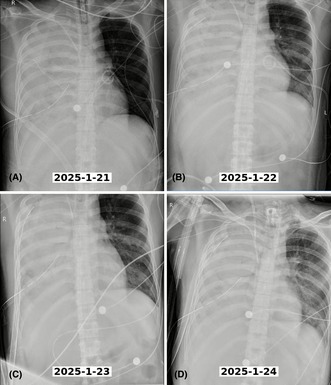

Extracorporeal cardiopulmonary resuscitation (ECPR) combined with emergent surgical repair can achieve hemodynamic stabilization in selected patients with penetrating chest trauma and thoracic aortic injury complicated by cardiac arrest. Early initiation and rapid hemorrhage control are critical determinants of outcome.

## 1 Introduction

Penetrating chest trauma with thoracic aortic injury is a life‐threatening emergency with extremely high mortality rates, with reported pre‐hospital mortality exceeding 80% and overall survival rates below 20% in most series [1, 2]. When complicated by cardiac arrest, the prognosis is particularly poor with conventional cardiopulmonary resuscitation (CPR) alone, with survival rates approaching zero without definitive hemorrhage control [1, 2]. Extracorporeal membrane oxygenation (ECMO) refers to a life support technique that uses a pump and membrane oxygenator to provide gas exchange and circulatory support outside the body. Veno‐arterial ECMO (VA‐ECMO) provides both cardiac and respiratory support by draining deoxygenated blood from the venous system and returning oxygenated blood to the arterial system. Extracorporeal cardiopulmonary resuscitation (ECPR) has emerged as a promising therapeutic option for selected patients with refractory cardiac arrest [3, 4, 5]. ECPR refers specifically to the rapid initiation of VA‐ECMO during ongoing cardiac arrest to provide cardiopulmonary support while addressing the underlying cause. Ideal candidates for ECPR typically include patients with witnessed arrest, bystander CPR, presumed reversible etiology, age younger than 65–70 years, minimal comorbidities, and a no‐flow time < 5 min with low‐flow time < 60 min [3, 4]. However, major vascular injury has traditionally been considered a relative contraindication to ECPR in trauma patients primarily due to concerns about systemic anticoagulation requirements exacerbating hemorrhage and the theoretical risk of worsening bleeding from damaged vessels [6].

We present a case of successful ECPR treatment in a young patient with penetrating chest trauma and thoracic aortic injury, highlighting the importance of individualized patient selection, rapid multidisciplinary coordination, and the role of functional monitoring in guiding clinical management.

## 2 Case Report

### 2.1 Patient Information and Clinical Presentation

A 22‐year‐old unmarried male presented to our emergency department on January 17, 2025, at 11:54 following a stab wound to the chest sustained at 11:22. The patient had no significant past medical history and was previously healthy.

### 2.2 Timeline and Clinical Course

**FIGURE 1:**
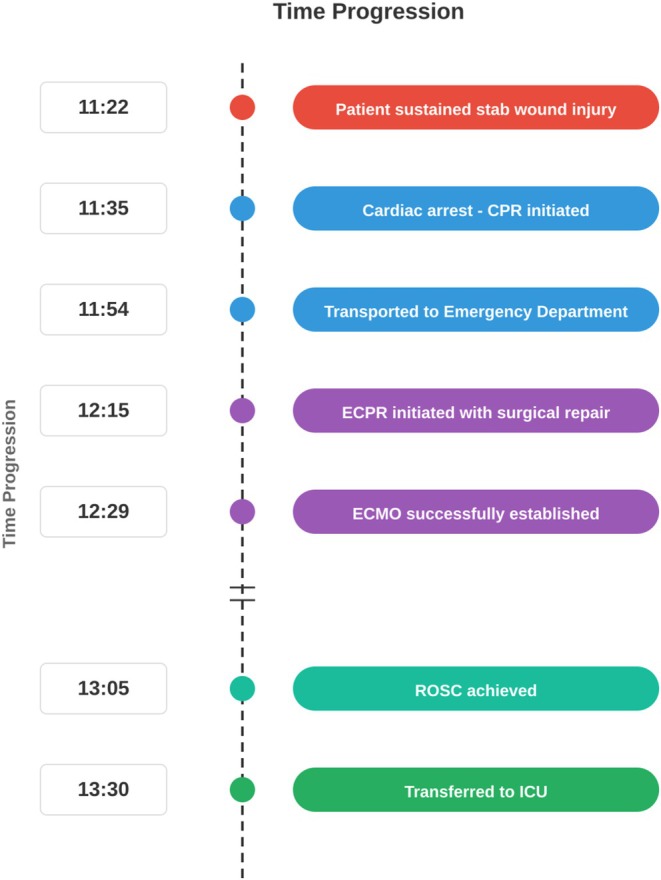
Timeline and clinical course. Schematic representation of the key events from initial injury through hospital course, including resuscitative thoracotomy, ECPR initiation, surgical interventions, and ECMO weaning milestones.

The clinical timeline is illustrated in Figure 1. At 11:22, the patient sustained a stab wound injury. Cardiac arrest occurred at 11:35, and CPR was initiated immediately. The patient was transported to our emergency department at 11:54 in ongoing cardiac arrest. Given the mechanism of injury and witnessed arrest with ongoing CPR, the decision was made to proceed with emergent resuscitative thoracotomy. A left anterolateral thoracotomy was performed at 12:15 for direct access to the heart and great vessels. Open cardiac massage was initiated while the source of hemorrhage was rapidly identified. ECPR was initiated concurrently with emergent surgical repair.

Surgical exploration revealed a 1.5 cm transverse laceration on the anterior wall of the ascending aorta (entry wound) and a 1.0 cm transverse laceration on the right posterior wall of the ascending aorta (exit wound). Both lacerations were repaired with continuous 4‐0 Prolene sutures. ECMO was successfully established at 12:29, and return of spontaneous circulation (ROSC) was achieved at 13:05. The patient was transferred to the intensive care unit at 13:30.

### 2.3 Diagnostic Assessment

Primary diagnoses included: cardiac arrest with successful resuscitation; penetrating chest trauma with thoracic aortic injury and sternal fracture; severe pneumonia with acute respiratory distress syndrome (ARDS); hemopneumothorax; multiple organ dysfunction syndrome (coagulopathy, hepatic dysfunction, gastrointestinal bleeding, cardiac dysfunction [Killip Class IV], hemopericardium); hemorrhagic shock with severe anemia; acute hypoxic–ischemic encephalopathy with brain contusion; electrolyte imbalance; and hypoproteinemia. Initial laboratory findings on admission (normal ranges in parentheses) demonstrated severe derangements consistent with hemorrhagic shock and tissue hypoperfusion: hemoglobin 5.2 g/dL (13.5–17.5 g/dL); hematocrit 16.3% (38.8%–50.0%); platelet count 89 × 10^9^/L (150–400 × 10^9^/L); INR 2.4 (0.9–1.1); fibrinogen 0.8 g/L (2.0–4.0 g/L); lactate 14.2 mmol/L (< 2.0 mmol/L); creatinine 168 μmol/L (62–106 μmol/L); ALT 892 U/L (7–56 U/L); AST 1245 U/L (10–40 U/L); total bilirubin 48 μmol/L (5.1–17.1 μmol/L); albumin 22 g/L (35–50 g/L); and troponin I 8.6 ng/mL (< 0.04 ng/mL). Arterial blood gas analysis revealed severe metabolic acidosis with respiratory compensation: pH 7.08 (7.35–7.45); PaCO2 28 mmHg (35–45 mmHg); PaO_2_ 52 mmHg on 100% FiO_2_; HCO3–8.2 mmol/L (22–26 mmol/L); base excess −19.4 mmol/L (−2 to +2 mmol/L). Over the subsequent days, laboratory parameters demonstrated gradual improvement correlating with clinical recovery. By Day 8 (ECMO decannulation), lactate had normalized to 1.4 mmol/L, hemoglobin stabilized at 10.2 g/dL with transfusion support, coagulation parameters corrected (INR 1.2), and liver enzymes showed a downward trend (ALT 245 U/L, AST 198 U/L).

### 2.4 Hospital Course

Day 1 (January 17, 2025): The patient remained critically ill in the ICU. Day 4 (January 20, 2025): Bedside ultrasound revealed massive hematoma in the right pleural cavity with diaphragmatic displacement and hepatic compression, accompanied by progressive elevation of liver enzymes. Day 5 (January 21, 2025): CT scan confirmed massive hematoma in the right pleural cavity, right lung atelectasis, hepatic compression and displacement, and diffuse cerebral edema with reduced brain tissue density consistent with hypoxic–ischemic encephalopathy.

Day 6 (January 22, 2025): Bedside thoracic hematoma evacuation was performed, with approximately 3000 mL of blood and clots removed. This resulted in relief of mediastinal and hepatic compression, progressive improvement in left lung function, and gradual reduction of ECMO parameters. Day 8 (January 24, 2025): Following multidisciplinary team evaluation, successful ECMO weaning was achieved after 8 days of support. The patient was hemodynamically stable. CT scan was repeated, tracheostomy was performed, and additional microbiological sampling was obtained.

### 2.5 Radiological Findings

**FIGURE 2:**
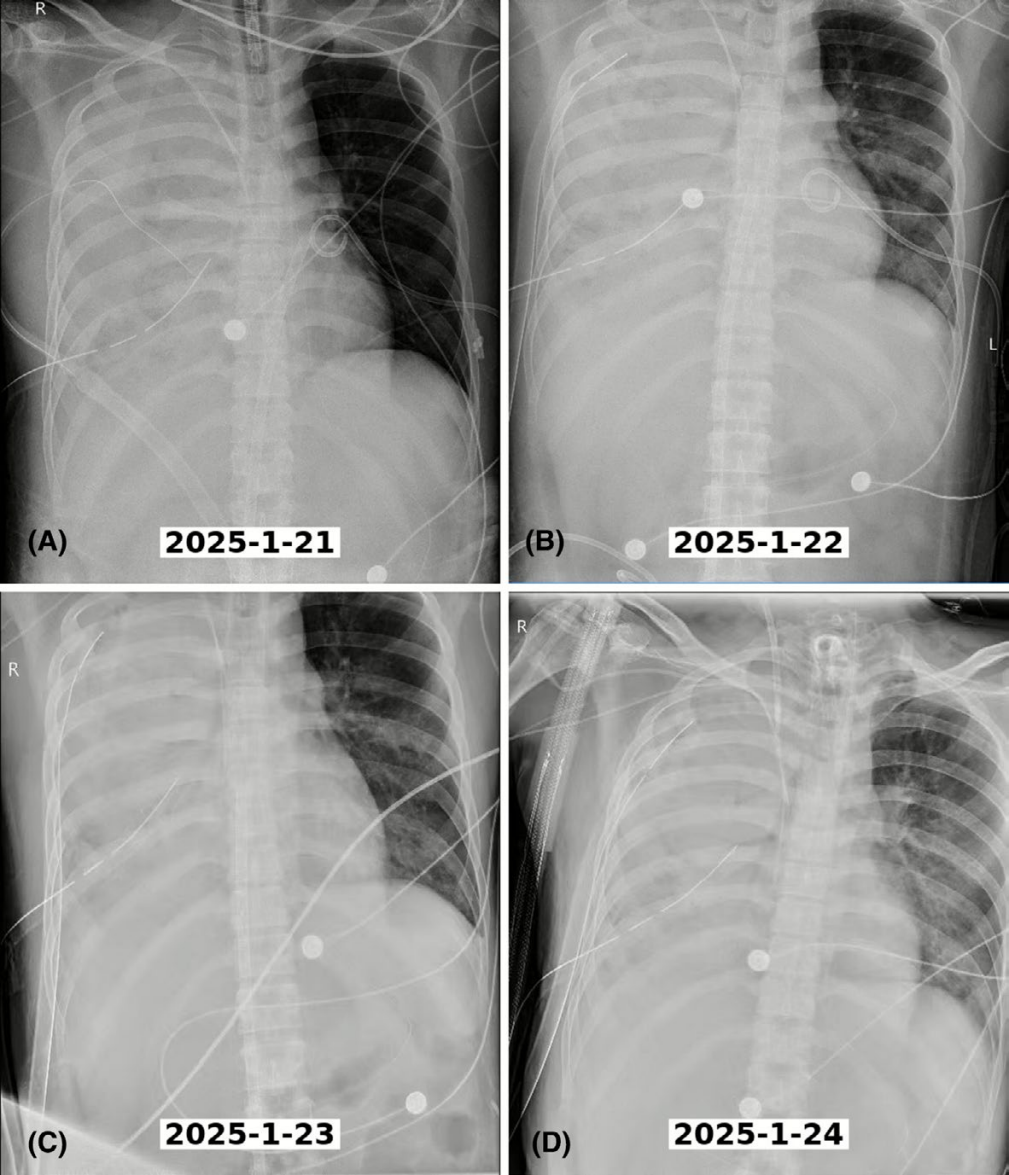
Serial Chest X‐rays Demonstrating Clinical Progression During ECPR Treatment. (A) January 21 (Day 5): Massive right hemothorax with complete right lung opacity and mediastinal shift to the left. (B) January 22 (Day 6): Immediate improvement following 3000 mL hematoma evacuation. (C) January 23 (Day 7): Progressive lung re‐expansion with decreasing pleural opacity. (D) January 24 (Day 8): Near‐complete resolution of hemothorax prior to successful ECMO decannulation.

Serial chest radiographs demonstrated dramatic recovery following ECPR intervention and surgical management (Figure 2). Day 5 (January 21) showed massive right hemothorax with complete lung collapse and severe mediastinal shift. Day 6 (January 22) demonstrated immediate improvement following bedside hematoma evacuation. Day 7 (January 23) showed progressive recovery with continued lung recruitment. Day 8 (January 24) documented near‐complete resolution with successful ECMO decannulation. The radiological progression correlated with improvement in PaO_2_/FiO_2_ ratio from 118 to 442.5.

**FIGURE 3:**
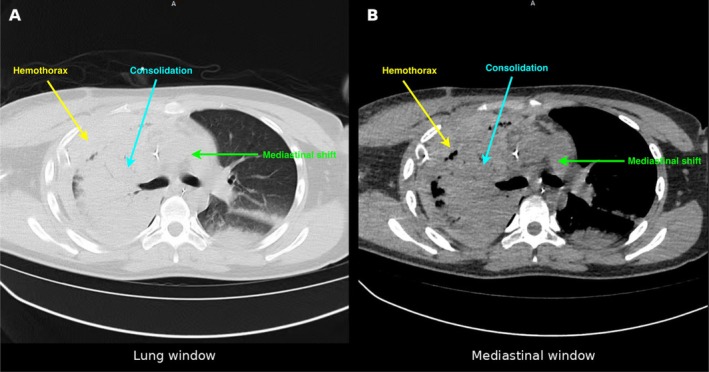
Chest CT on Day 5 (January 21, 2025). (A) Lung window showing massive right hemothorax with lung consolidation and mediastinal shift to the left. (B) Mediastinal window demonstrating the extent of pleural hematoma and hepatic compression. Yellow arrows indicate hemothorax; cyan arrows indicate consolidation; green arrows indicate mediastinal shift.

Computed tomography (CT) imaging on Day 5 (January 21, 2025) provided detailed assessment of intrathoracic pathology (Figure 3). Lung window images demonstrated massive right hemothorax with complete right lung atelectasis and significant mediastinal shift to the left. Mediastinal window images confirmed the extensive hematoma and revealed hepatic compression and displacement due to the mass effect of the pleural collection.

### 2.6 Electrical Impedance Tomography Monitoring

Regional lung ventilation and cardiac‐related impedance changes were monitored using electrical impedance tomography (EIT). EIT provides functional imaging of regional lung ventilation (V), perfusion‐related impedance changes (P), and their combined distribution (C). EIT‐derived “perfusion” represents cardiac‐synchronous impedance variations reflecting cyclic changes in regional pulmonary blood volume, rather than direct measurements of absolute pulmonary blood flow.

**FIGURE 4:**
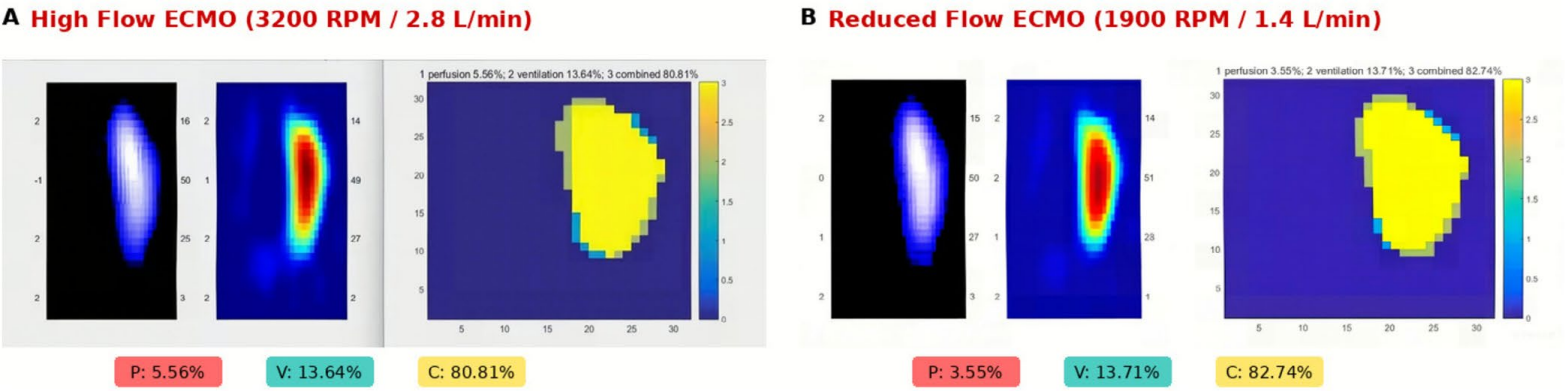
EIT Monitoring During VA‐ECMO Flow Adjustment. (A) High flow settings (3200 RPM, 2.8 L/min) showing perfusion 5.56%, ventilation 13.64%, and combined index 80.81%. (B) Reduced flow settings (1900 RPM, 1.4 L/min) showing perfusion decreased to 3.55% while ventilation remained stable at 13.71%, with combined index 82.74%. Each panel displays impedance‐derived perfusion map (left), ventilation map (center), and V/Q index distribution (right). C, combined index; *P*, perfusion; V, ventilation.

During VA‐ECMO flow adjustment (Figure 4), high‐flow settings (3200 RPM/2.8 L/min) demonstrated perfusion of 5.56%, ventilation of 13.64%, and combined index of 80.81% with predominant left lung ventilation. Following 50% flow reduction (1900 RPM/1.4 L/min), perfusion decreased to 3.55% while ventilation remained stable at 13.71%, with minimal change in right lung recruitment, demonstrating that flow adjustment alone was insufficient for right lung recruitment.

**FIGURE 5:**
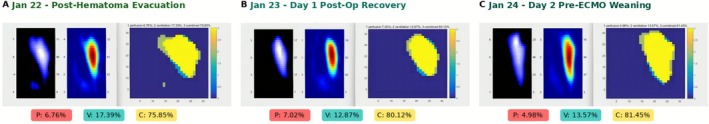
EIT Monitoring of Post‐Surgical Recovery Timeline. (A) January 22 (Post‐Hematoma Evacuation): Immediate effect of 3000 mL hematoma evacuation showing perfusion 6.76%, ventilation 17.39%, and combined index 75.85%. (B) January 23 (Day 1 Post‐Operative): Stabilization with perfusion 7.02%, ventilation 12.87%, and combined index 80.12%. (C) January 24 (Day 2 Pre‐ECMO Weaning): Optimal state achieved with perfusion 4.98%, ventilation 13.57%, and combined index 81.45%, indicating readiness for ECMO weaning. C, combined index; *P*, perfusion; V, ventilation.

Post‐surgical recovery monitoring with EIT (Figure 5) demonstrated rapid lung recruitment following 3000 mL hematoma evacuation. Regional ventilation distribution improved markedly from 13.64% to 17.39% immediately post‐evacuation, with progressive normalization of functional ventilation‐perfusion matching. Final measurements on Day 2 showed favorable functional status supporting successful ECMO weaning.

### 2.7 ECMO Parameters

ECMO parameters and respiratory variables during the 8‐day support period are summarized in Table 1. The progressive improvement in PaO_2_/FiO_2_ ratio (from 118 on Day 1 to 442.5 on Day 8) reflects the combined effect of surgical intervention, hematoma evacuation, and optimized ventilatory support.

**TABLE 1:** ECMO parameters and respiratory variables during 8‐day support period.

Parameter	Day 1	Day 2	Day 3	Day 4	Day 5	Day 6	Day 7	Day 8
Speed (rpm)	3283	3110	3143	3142	3014	3055	3055	2210
Flow (L/min)	3.05	3.52	3.2	3.27	2.93	2.72	2.65	2
Gas Flow (L/min)	10	6	5	5	5	7	5	2
FiO_2_ (%)	60	50	50	70	60	60	50	40
Ventilator mode	V/C	PRVC	PRVC	PRVC	PRVC	PRVC	PRVC	PRVC
Ventilator FiO_2_ (%)	65	80	70	75	60	55	55	55
Tidal volume (mL)	300	150	200	200	280	350	400	400
PEEP (cmH_2_O)	13	16	15	12	12	13	10	10
PaO_2_/FiO_2_	118	96.25	188.57	208.57	263.33	245.71	395	442.5

Abbreviations: PaO_2_/FiO_2_, arterial oxygen tension to fractional inspired oxygen ratio; PEEP, positive end‐expiratory pressure; PRVC, pressure‐regulated volume control; V/C, volume control.

### 2.8 Neurological Outcome

Due to prolonged cardiac arrest time exceeding 30 min (approximately 50 min from arrest to ROSC), the patient did not achieve neurological recovery. On admission, the Glasgow Coma Scale (GCS) score was 3T (intubated, no eye opening, no verbal response, no motor response). Despite successful ECMO weaning and hemodynamic stabilization, the GCS remained at 3T throughout the hospital course. Day 5 CT imaging demonstrated diffuse cerebral edema with reduced brain tissue density consistent with severe hypoxic–ischemic encephalopathy. Serial neurological assessments showed no improvement in consciousness level. After extensive discussions regarding the poor neurological prognosis and quality of life considerations, the patient's family decided to withdraw further life‐sustaining treatment approximately 2 months after the initial injury. This outcome underscores the critical importance of minimizing arrest‐to‐ECPR time in determining neurological outcomes in traumatic cardiac arrest.

## 3 Discussion

This case illustrates several critical aspects of managing severe penetrating chest trauma with cardiac arrest. The successful hemodynamic stabilization depended on multiple factors: rapid recognition and response with early identification of cardiac arrest and immediate CPR initiation; a multidisciplinary approach involving coordinated care from emergency medicine, cardiothoracic surgery, and intensive care teams; timely surgical intervention with prompt thoracotomy and direct repair of aortic injuries; strategic ECPR implementation during the most critical phase of resuscitation; multimodal monitoring integrating conventional hemodynamic parameters, serial imaging, and functional assessment tools; and proactive complication management [7, 8, 9, 10].

ECPR provided essential cardiopulmonary support during the critical window for definitive surgical repair, demonstrating its potential role in selected traumatic cardiac arrest patients with surgically correctable injuries [11, 12]. Comparing our case to previously reported series provides important context. Dennis et al. [11] reported on a multicentre ECPR experience that included trauma patients, noting survival rates of approximately 30% in carefully selected cases, with younger age and witnessed arrest being favorable prognostic factors. Similarly, Spillner et al. [12] described outcomes from emergency ECPR and identified that patients with potentially reversible causes, particularly those amenable to surgical correction, had significantly better outcomes than those with medical cardiac arrests. Our patient shared several favorable characteristics with survivors in these series: young age (22 years), witnessed arrest with immediate bystander CPR, and a surgically correctable injury. However, the prolonged no‐flow and low‐flow time (approximately 50 min total) exceeded the typically recommended thresholds for favorable neurological outcomes. The key technical success was the concurrent approach of hemorrhage control during ECPR initiation rather than sequential management. The massive hemothorax created a compressive physiology that prevented lung recruitment despite maximal ECMO support. Bedside hematoma evacuation proved to be the critical intervention, with immediate improvement in both ventilation distribution and oxygenation parameters.

EIT monitoring provided real‐time functional assessment of regional lung mechanics, offering mechanistic insights into treatment response [13, 14]. While EIT‐derived indices represent impedance‐based surrogates rather than absolute hemodynamic measurements, they successfully identified the lack of response to ECMO flow adjustment alone and documented the dramatic effect of mechanical decompression. This underscores the primacy of addressing the underlying injury over purely supportive measures in trauma ECPR.

The unfavorable neurological outcome in this case highlights the critical importance of arrest time in determining prognosis. While ECPR successfully achieved hemodynamic stabilization and allowed for definitive surgical repair of the aortic injury, the prolonged arrest duration resulted in irreversible hypoxic–ischemic brain injury. This case suggests that in similar scenarios with shorter arrest‐to‐ECPR intervals, favorable neurological outcomes may be achievable. The experience gained from this case provides valuable insights for the management of future patients presenting with penetrating chest trauma and cardiac arrest.

This case challenges the conventional wisdom that major vascular injury should be an absolute contraindication to ECPR. In young patients with witnessed arrest, immediate CPR, and surgically correctable injuries, ECPR may provide a viable rescue option. Patient selection should be individualized, with particular attention to minimizing arrest‐to‐ECPR time to optimize neurological outcomes.

## 4 Conclusion

This case demonstrates the technical feasibility of ECPR in treating severe penetrating chest trauma with thoracic aortic injury complicated by cardiac arrest. The integration of immediate surgical intervention, ECPR support, and comprehensive multidisciplinary care achieved successful hemodynamic stabilization and ECMO weaning. However, the prolonged arrest time (> 30 min) resulted in an unfavorable neurological outcome. While EIT provided valuable functional insights for guiding respiratory management, the case underscores that minimizing arrest‐to‐ECPR time is paramount for neurological recovery. This experience supports the inclusion of ECPR as a viable rescue option in treatment protocols for selected patients with traumatic cardiac arrest and surgically correctable injuries and provides valuable reference for managing similar cases with potentially better outcomes when shorter arrest intervals can be achieved.

## Author Contributions

**Xiang Gao:** resources, supervision. **Linzi Jin:** data curation, formal analysis. **Yan Li:** writing – review and editing.

## Funding

The authors have nothing to report.

## Ethics Statement

Written informed consent was obtained from the patient for publication of this case report and any accompanying images.

## Conflicts of Interest

The authors declare no conflicts of interest.

## Data Availability

The data that support the findings of this study are available from the corresponding author upon reasonable request.
